# Electronic Tuning of CO_2_ Interaction by Oriented Coordination of N‐Rich Auxiliary in Porphyrin Metal–Organic Frameworks for Light‐Assisted CO_2_ Electroreduction

**DOI:** 10.1002/advs.202301261

**Published:** 2023-05-01

**Authors:** Zhifeng Xin, Xue Dong, Yi‐Rong Wang, Qian Wang, Kejing Shen, Jing‐Wen Shi, Yifa Chen, Ya‐Qian Lan

**Affiliations:** ^1^ Institute of Molecular Engineering and Applied Chemistry Anhui University of Technology Ma'anshan Anhui 243002 P. R. China; ^2^ National and Local Joint Engineering Research Center of MPTES in High Energy and Safety LIBs Engineering Research Center of MTEES (Ministry of Education) Key Laboratory of ETESPG (GHEI) School of Chemistry South China Normal University Guangzhou 510006 P. R. China

**Keywords:** CO_2_ electroreduction, CO_2_ interaction, hexamethylene tetramine, metal–organic framework, oriented coordination

## Abstract

The efficient CO_2_ electroreduction into high‐value products largely relies on the CO_2_ adsorption/activation or electron‐transfer of electrocatalysts, thus site‐specific functionalization methods that enable boosted related interactions of electrocatalysts are much desired. Here, an oriented coordination strategy is reported to introduce N‐rich auxiliary (i.e., hexamethylenetetramine, HMTA) into metalloporphyrin metal organic frameworks (MOFs) to synthesize a series of site‐specific functionalized electrocatalysts (HMTA@MOF‐545‐M, M = Fe, Co, and Ni) and they are successfully applied in light‐assisted CO_2_ electroreduction. Noteworthy, thus‐obtained HMTA@MOF‐545‐Co presents approximately two times enhanced CO_2_ adsorption‐enthalpy and electrochemical active surface‐area with largely decreased impedance‐value after modification, resulting in almost twice higher CO_2_ electroreduction performance than its unmodified counterpart. Besides, its CO_2_ electroreduction performance can be further improved under light‐illumination and displays superior FE_CO_ (≈100%), high CO generation rate (≈5.11 mol m^−2^ h^−1^ at −1.1 V) and energy efficiency (≈70% at −0.7 V). Theoretical calculations verify that the oriented coordination of HMTA can increase the charge density of active sites, almost doubly enhance the CO_2_ adsorption energy, and largely reduce the energy barrier of rate determining step for the boosted performance improvement. This work might promote the development of modifiable porous crystalline electrocatalysts in high‐efficiency CO_2_ electroreduction.

## Graphical abstract

A kind of N‐rich auxiliary (i.e., hexamethylenetetramine, HMTA) is successfully introduced into metalloporphyrin metal organic frameworks (MOFs) to synthesize a series of site‐specific functionalized electrocatalysts (HMTA@MOF‐545‐M, M = Fe, Co, and Ni) that can be successfully applied in efficient light‐assisted CO_2_ electroreduction.

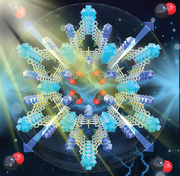

## 1 Introduction

With the increasing concern about the protection of human ecological environment and global climate, CO_2_ emission caused by the development of industry have aroused to be a worldwide issue owing to its serious impact on the environment.^[^ ^1^, ^2^ ^]^ To reduce the CO_2_ emission, a series of technologies have been developed,^[^ ^3^ ^]^ including carbon capture and storage^[^ ^4^, ^5^ ^]^ or carbon capture and utilization (CCU),^[^ ^6^, ^7^ ^]^ etc. In particular, the CCU technology that can convert CO_2_ into valuable chemicals seems to be more attractive, in which the notorious CO_2_ can be upgraded into the market‐value products with a broad range of application fields (e.g., biological, medical, and industrial fields, etc.).^[^ ^8^, ^9^, ^10^, ^11^ ^]^ At present, except for the traditional CCU technology, some advanced techniques such as photocatalytic CO_2_ reduction reaction,^[^ ^12^, ^13^ ^]^ CO_2_ bioconversion,^[^ ^14^ ^]^ or electrocatalytic CO_2_ reduction reaction (CO_2_RR)^[^ ^15^ ^]^ have been intensively studied during past decades.^[^ ^15^, ^16^ ^]^ Among them, electrocatalytic CO_2_RR has attracted extensive attention due to its possibility in transformation of CO_2_ into serviceable high‐valued chemicals or fuels (e.g., CO, HCOOH, CH_4_, C_2_H_4_, C_2_H_2_, CH_3_COOH, C_2_H_5_OH, etc.) by renewable electricity.^[^ ^17^, ^18^ ^]^ However, the electrocatalytic CO_2_RR is still obstructed by the competing thermodynamically favorable hydrogen evolution reaction and multiple proton‐coupled electron transfer processes during the CO_2_RR process, in which different conversion pathways will occur under similar reduction potentials and result in relatively low product selectivity.^[^ ^19^ ^]^ Besides, traditional electrocatalytic CO_2_RR processes have also been restricted by the current equipment or techniques, which needs more advanced strategies to promote the development of electrocatalytic CO_2_RR.^[^ ^20^ ^]^ In this regard, it will be much necessary to design highly active catalysts and develop advanced techniques to achieve efficient electrocatalytic CO_2_RR for its potentially industrial applications.

During past years, various kinds of materials have been developed as electrocatalysts in this field,^[^ ^21^ ^]^ including transition metal oxides,^[^ ^22^ ^]^ transition metal chalcogenides,^[^ ^23^ ^]^ metal‐free 2D materials,^[^ ^10^, ^24^ ^]^ porous organic polymers,^[^ ^25^ ^]^ covalent organic frameworks (COFs),^[^ ^26^ ^]^ metal organic frameworks (MOFs),^[^ ^27^, ^28^, ^29^ ^]^ etc. Among them, MOFs are a new type of porous crystalline materials constructed by metal ions and organic ligands, which possess predesignable and atomically ordered structures, large surface area and tunable functionality that have much potential in electrocatalytic CO_2_RR.^[^ ^30^, ^31^, ^32^ ^]^ However, the crystalline nature has set bottlenecks of MOFs in this field, including the generally low electron transfer efficiency or insufficient CO_2_ adsorption/activation property, thus largely restricting the catalytic efficiency of MOFs to satisfy the needs of potentially practical applications. To maximize the advantages of MOFs and overcome their shortcomings, diverse postmodification methods have been developed to improve their catalytic performances (e.g., loading of conductive agents or hybridization with carbon materials,^[^ ^33^ ^]^ doping electron rich clusters,^[^ ^34^, ^35^ ^]^ introducing functional groups,^[^ ^19^ ^]^ etc.) in addition to the basic structure design.^[^ ^36^ ^]^ Upon tuning the specific functions around the active sites, the CO_2_ interaction as well as CO_2_RR properties of MOFs might be simultaneously improved. Nevertheless, the electronic tuning of CO_2_ adsorption/activation interaction with MOFs, a vital and perquisite factor for improving the electrocatalytic CO_2_RR performance, has been pained less attention in this field to the best of our knowledge. Thus, powerful methods that can achieve the boosted CO_2_ interaction with MOFs to largely facilitate their electrocatalytic CO_2_RR performances would be much desirable.

In general, CO_2_ as a kind of notorious and acidic gas is thermodynamically stable and chemically inert due to the high bonding energy (750 kJ mol^−1^) of C=O bond.^[^ ^37^ ^]^ To improve the adsorption or activation interaction with CO_2_ molecule, the introduction of functional or alkaline auxiliaries into the porous MOFs structures will be much necessary to conquer the bottlenecks of MOFs in CO_2_ interaction and further electroreduction into more valuable products.^[^ ^38^, ^39^ ^]^ As one of the potential and promising postmodification agent, hexamethylenetetramine (HMTA) is a kind of alkaline tetradentate molecule with adamantane structure that composed of four nitrogen atoms and six carbon atoms, in which the abundant nitrogen coordination sites can anchor both the metal sites and CO_2_ molecules from two directions.^[^ ^40^ ^]^ Doping HMTA molecules into MOFs channels might improve the CO_2_ adsorption capacity of MOFs, and effectively combine the characteristics of Lewis bases and MOFs structures to improve the CO_2_ interaction and further electrocatalytic CO_2_RR efficiency.^[^ ^41^ ^]^ To match the structure of HMTA, the target MOFs will preferably possess unsaturated metal sites containing an empty orbital to accept the lone pair electrons of HMTA. With these considerations in mind, metalloporphyrin based MOFs, a kind of promising CO_2_RR electrocatalysts in generation of valuable products, come to our mind.^[^ ^42^, ^43^ ^]^ Specifically, metalloporphyrin based MOFs with abundant and modifiable metalloporphyrin centers are candidate materials to be cooperated with HMTA due to the empty orbitals existed in metal sites of metalloporphyrin rings, which can accept the lone pair electrons of HMTA and create powerful HMTA modified MOFs electrocatalysts. From the above we deduce that HMTA modified metalloporphyrin MOFs might probably be the promising candidates to enhance the efficiency of CO_2_RR, while the assembly of such MOFs hybrid system with site‐specific functionality is still rare and unmet as far as we know.

**Scheme 1:**
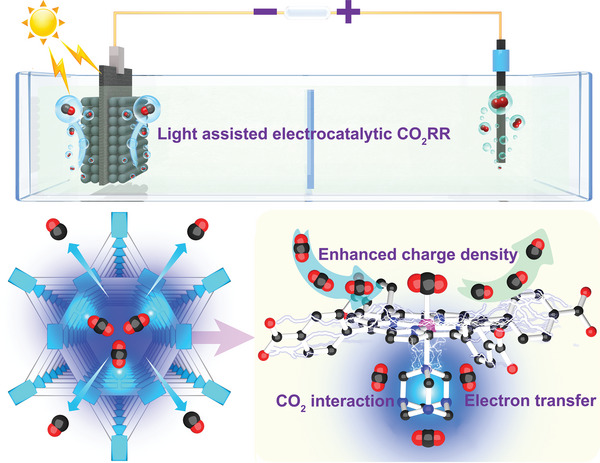
The schematic illustration of HMTA implanted MOF‐545‐M for light‐assisted electrocatalytic CO_2_RR.

Herein, we develop an oriented coordination strategy to introduce N‐rich auxiliary (i.e., HMTA) into metalloporphyrin MOFs to synthesize a series of site‐specific functionalized electrocatalysts (HMTA@MOF‐545‐M, M = Fe, Co, and Ni) that can be successfully applied in efficient light‐assisted CO_2_ electroreduction (**Scheme** **1**). After modification, HMTA can be coordinated with the metalloporphyrin centers in the channels of MOF‐545‐M (M = Fe, Co, and Ni) and induced strong interaction with CO_2_ molecules. Specifically, thus‐obtained HMTA@MOF‐545‐Co presents approximately two times enhanced CO_2_ adsorption‐enthalpy and electrochemical active surface‐area with largely decreased impedance‐value after modification, resulting in almost twice higher CO_2_ electroreduction performance than its unmodified counterpart. Besides, the CO_2_ electroreduction performance can be further improved under visible light‐illumination. Theoretical calculation verifies proves that the oriented coordination of HMTA can facilitate the CO_2_ adsorption/activation, increase the charge density of active sites, and largely reduce the energy barrier of rate‐determining step to improve the electrocatalytic CO_2_RR performance.

## 2 Results and Discussion

### 2.1 Synthesis and Characterization

**Figure 1:**
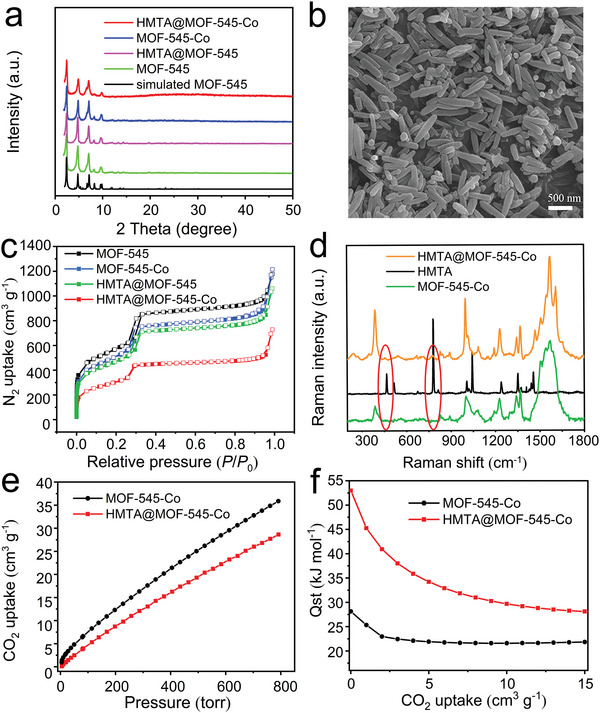
Characterization of HMTA@MOF‐545‐Co. a) PXRD patterns of HMTA@MOF‐545‐Co, MOF‐545‐Co, HMTA@MOF‐545, MOF‐545, and simulated MOF‐545. b) SEM image of HMTA@MOF‐545‐Co. c) N_2_ sorption isotherms of HMTA@MOF‐545‐Co, MOF‐545‐Co, HMTA@MOF‐545, and MOF‐545. d) Raman spectra of HMTA@MOF‐545‐Co, MOF‐545‐Co, and HMTA. e) CO_2_ adsorption curves of HMTA@MOF‐545‐Co and MOF‐545‐Co tested at 298 K. f) CO_2_ adsorption enthalpy of HMTA@MOF‐545‐Co and MOF‐545.

For the preparation of HMTA@MOF‐545‐M (M = Fe, Co, and Ni), MOF‐545‐M (M = Fe, Co, and Ni) are first prepared by the solvothermal reactions of MOF‐545 and related metal salts.^[^ ^33^, ^34^ ^]^ Then, HMTA@MOF‐545‐M (M = Fe, Co, and Ni) are further synthesized by the reaction of HMTA and MOF‐545‐M (M = Fe, Co, and Ni) through a facile sonication method using chloroform as the solvent at 40 °C (detail see the Experimental Section). To characterize them, the powder X‐ray diffraction (PXRD) tests are first conducted. Taking HMTA@MOF‐545‐Co as an example, the experimental result is consistent with the simulated data of MOF‐545‐Co, indicating that the implantation of HMTA has not changed the inherent structure of MOF‐545‐Co (**Figure** **1**a). For HMTA@MOF‐545‐Fe and HMTA@MOF‐545‐Ni, they also display similar PXRD patterns to their unmodified counterparts (Figure S1, Supporting Information).

In addition, Fourier Transform infrared (FT‐IR) tests have been carried out to characterize the components of the samples. Specifically, the vibration peak of M—N bond is found at about 1000 cm^−1^ in the FT‐IR spectrum, indicating that the metals in MOF‐545‐M (M = Fe, Co, and Ni) coordinate with the N sites of meso‐tetra(4‐carboxyphenyl) porphyrin (TCPP) (Figure S2a, Supporting Information).^[^ ^44^ ^]^ Specifically, the FT‐IR spectra of HMTA@MOF‐545‐Co show two strong peaks at 1000 and 1475 cm^−1^ that can be ascribed to the C—N and CH_2_ stretching vibration of HMTA, respectively, indicating the presence of HMTA in the hybrid material (Figure S2b, Supporting Information). Besides, a slightly split peak at about 1020 cm^−1^ verifies the coordination of N in HMTA with Co site according to the literatures.^[^ ^45^, ^46^ ^]^ To better confirm the presence of HMTA in HMTA@MOF‐545‐Co, Raman tests have been further carried out (Figure 1d). Raman spectroscopy is a sensitive characterization technology to study the weak polar bond and it is complementary to FT‐IR investigation. The peaks at 1040 and 462 cm^−1^ are attributed to the stretching vibration of N—C—N bond and the peak at 777 cm^−1^ would be the N—C stretching mode of HMTA,^[^ ^47^, ^48^ ^]^ in which the N—C—N bending modes possess the symmetries E and the C—N stretching modes possess the symmetries A1, respectively. Compared with the characteristic peaks of MOF‐545‐Co, no such peaks are observed. The result indicates that HMTA is successfully introduced in the structure of HMTA@MOF‐545‐Co. Besides, the X‐ray photoelectron spectroscopy (XPS) tests have been performed to illustrate the elemental state and bonding energy of samples (Figure S3, Supporting Information). As depicted in the high resolution XPS spectra, the N 1s peak of HMTA@MOF‐545‐Co (398.97 eV) has a ≈0.2 eV decrease when compared with that of MOF‐545‐Co (399.18 eV). Meanwhile, as detected, the Co 2p_3/2_ (781.79 eV) and Co 2p_1/2_ (797.46 eV) of HMTA@MOF‐545‐Co have positive shifts of ≈0.2 and ≈0.3 eV in comparison with those of MOF‐545‐Co (i.e., Co 2p_3/2_ (781.59 eV) and Co 2p_1/2_ (797.17 eV)), respectively. These results indicate that the lost valence electrons might be possibly transferred from Co center to N sites of HMTA after modification.^[^ ^49^, ^50^ ^]^

As expected, scanning electron microscope (SEM) images reveal that the as‐prepared HMTA@MOF‐545‐Co can maintain the original rod‐like structure of MOF‐545‐Co (Figure 1b and Figure S4, Supporting Information). Besides, the smooth surface of HMTA@MOF‐545‐Co certifies that no HMTA particles recrystallized on the surface of MOF‐545‐Co. Similar results have been observed in the SEM images of HMTA@MOF‐545‐Fe and HMTA@MOF‐545‐Ni (Figure S5, Supporting Information). In addition, transmission electron microscope (TEM) (Figure S6a, Supporting Information) of HMTA@MOF‐545‐Co also proves the results detected in the SEM tests, and the high‐resolution TEM image (Figure S6b, Supporting Information) indicates that no HMTA aggregated in the pore of MOF‐545‐Co. Moreover, the energy dispersive X‐ray spectroscopy (EDS) elemental mapping characterization shows that C, N, O, Zr, and Co elements are homogeneously dispersed in HMTA@MOF‐545‐Co, verifying the uniform dispersion of HMTA in the pore channels (Figure S7, Supporting Information).

Besides, the porosity of the obtained samples has been measured based on the N_2_ sorption tests at 77 K (Figure 1c). Taking the HMTA@MOF‐545‐Co for instance, the Brunauer–Emmett–Teller surface area (*S*_BET_) of MOF‐545, MOF‐545‐Co, HMTA@MOF‐545, and HMTA@MOF‐545‐Co are calculated to be 2023, 1957, 1730, and 1316 m^2^ g^−1^, respectively. Besides, the centered pore size in the pore size distribution curves slightly decreases from 2.6 to 2.4 nm (Figure S8 and Table S1, Supporting Information). Thus, the modification of HMTA molecular can decrease the pore volume and *S*_BET_ to some extend (Figure S8, Supporting Information), which can further verify that HMTA is successfully doped into the pore channels of MOF‐545‐Co. Based on the above results, we can conclude that the successful modification of HMTA in the MOF structure, in which the Zr_6_O_8_ clusters are linked by M‐TCPP ligand to assemble the porous MOF structure while HMTA loads into the pore channel and connects with the metal center.^[^ ^42^, ^43^ ^]^

To further investigate the adsorption performance of the HMTA modified MOFs, CO_2_ adsorption measurements are evaluated at 298, 283, and 273 K. The CO_2_ adsorption capacity of HMTA@MOF‐545‐Co (28.6 cm^3^ g^−1^) is slightly lower than that of MOF‐545‐Co (35.9 cm^3^ g^−1^) at 298 K and 1 atm (Figure 1e). The decrease of CO_2_ uptake will be attributed to the loading of HMTA molecular that occupies partial pore volume of MOFs. Furthermore, the CO_2_ adsorption capacity of HMTA@MOF‐545‐Co increases with the decrease of temperature (e.g., 44.7 cm^3^ g^−1^ at 283 K and 59.1 cm^2^ g^−1^ at 273 K) (Figure S9, Supporting Information). Meanwhile, the CO_2_ adsorption enthalpies of HMTA@MOF‐545‐Co and MOF‐545‐Co are calculated based on the CO_2_ uptake results obtained at 273, 283, and 298 K. Significantly, the CO_2_ adsorption enthalpy of HMTA@MOF‐545‐Co (52.98 kJ mol^−1^) is almost doubly enhanced than that of MOF‐545‐Co (28.10 kJ mol^−1^) (Figure 1f). The largely enhanced adsorption enthalpy might be attributed to the introduction of HMTA that provides more Lewis base sites to be favorable for the interaction with CO_2_, which also might set fundamental basis for the investigation of electrocatalytic CO_2_RR performance.

To enrich the diversity of samples, various loadings of HMTA have also been studied. Taking HMTA@MOF‐545‐Co for instance, HMTA@MOF‐545‐Co with different HMTA loadings can be prepared through tuning the adding amount of HMTA (e.g., 134, 67, and 33.5 mg HMTA) in the precursors (details see the Experimental Section) and these prepared samples with retained inert structures have been characterized by PXRD tests (Figure S10a, Supporting Information). Thermogravimetric analysis (TGA) measurement is used to detect the loading amount of HMTA in the structure of MOF‐545‐Co owing to the boiling point of HMTA (i.e., 263 °C) and the decomposition temperature of MOF‐545‐Co (i.e., ≈400 °C) is quite different (Figure S10b, Supporting Information). Before testing the TGA analysis, all the samples are degassed at 120 °C under dynamic vacuum for 8 h to ensure all the solvents in the pore of MOF are removed. Thus, two weight loss platforms can be detected in the TGA curves: the weight loss before 400 °C can be attributed to the loss of HMTA and the weight loss after 400 °C can be ascribed to the structure decomposition of MOF‐545‐Co. Based on the calculation results, the loading ratios of HMTA to Co‐TCPP in HMTA@MOF‐545‐Co are detected to be about 1:1, 1:2, and 3:4, respectively. Furthermore, the loading amount of HMTA has also been detected by elemental analysis and EDS characterizations and these results are basically consistent with the calculated values from TGA analysis (Figure S11 and Table S2, Supporting Information).

### 2.2 The Electrochemical Performances

**Figure 2:**
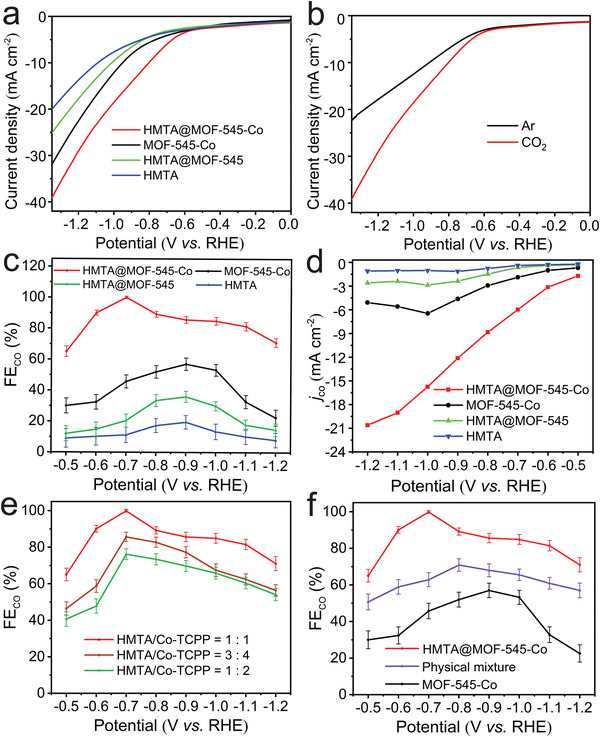
Electrocatalytic CO_2_RR performances. a) LSV curves of HMTA@MOF‐545‐Co and comparison sample. b) LSV curves of HMTA@MOF‐545‐Co in Ar and CO_2_ saturated 0.5 mol L^−1^ KHCO_3_ aqueous solution. c) Faradaic efficiencies for CO at different potentials. d) Partial CO current density. e) FE_CO_ of HMTA@MOF‐545‐Co with different HMTA loading capacity. f) FE_CO_ of HMTA@MOF‐545‐Co, MOF‐545‐Co, and physical mixture (HMTA + MOF‐545‐Co).

To investigate the effect of HMTA loading on the electrocatalytic CO_2_RR performance, HMTA@MOF‐545‐Co containing different HMTA loadings (e.g., HMTA/Co‐TCPP = 1:1, 1:2, and 3:4) have been investigated. As shown in **Figure** **2**e, the FE_CO_ of HMTA@MOF‐545‐Co (HMTA/Co‐TCPP = 1 : 1) is higher than that of the other two proportions (i.e., HMTA/Co‐TCPP = 1:2 and 3:4). Concretely, the FE_CO_ of HMTA@MOF‐545‐Co with HMTA/Co‐TCPP = 1:1 (≈100.0%, −0.7 V) is higher than other two HMTA loadings (i.e., HMTA/Co‐TCPP = 1:2, 76.2% and HMTA/Co‐TCPP = 3:4, 85.6%, at −0.7 V), indicating that the load capacity of HMTA/Co‐TCPP = 1:1 to be the optimal HMTA loading. Thus, we select HMTA@MOF‐545‐Co with the optimal HMTA loading as the model sample to further investigate the electrocatalytic CO_2_RR property.

In addition, the catalytic activity of HMTA@MOF‐545‐Co is evaluated using linear sweep voltammetry (LSV) in 0.5 mm KHCO_3_ electrolyte under CO_2_ saturation. The LSV curves of HMTA@MOF‐545‐Co show lower onset potential and higher current density than those of MOF‐545‐Co, MOF‐545, and HMTA@MOF‐545 (Figure 2a). Besides, compared with the LSV curve measured in Ar saturated KHCO_3_ solution, the current density measured in CO_2_ saturated electrolyte is much higher, indicating the increase of current comes from the reaction of CO_2_RR (Figure 2b). The reduction products are measured by gas chromatography (GC) and ^1^H nuclear magnetic resonance (^1^H NMR). Specifically, ^13^CO_2_ is used as the gas substrate in CO_2_RR process and the product is detected by gas chromatography mass spectra to define the product resource. The mass spectrum (Figure S12a, Supporting Information) shows that the peak at *m*/z = 29 is ascribed to ^13^CO, which confirms that the produced CO indeed comes from electrocatalytic CO_2_RR. Besides, the GC result exhibits that CO is the main gas product, and no liquid product is detected in ^1^H NMR tests (Figure S12b, Supporting Information).

Apart from that, the electrocatalytic CO_2_RR performances of HMTA@MOF‐545‐M (M = Fe and Ni) and their counterparts have also been evaluated (Figure S13a,b, Supporting Information). The results indicate that the FE_CO_ value of MOF‐545‐Co (56.99%, −0.9 V) is higher than both MOF‐545‐Fe (47.68%, −0.8 V) and MOF‐545‐Ni (40.78%, −1.0 V), which is consistent with the previously reported results for metalloporphyrin based MOFs.^[^ ^33^, ^34^, ^35^ ^]^ Simultaneously, the FE_CO_ of HMTA@MOF‐545‐Fe (82.91%, −0.8 V) and HMTA@MOF‐545‐Ni (79.47%, −0.9 V) are lower than that of HMTA@MOF‐545‐Co (≈100%, −0.70 V) (Figure 2c and Figure S13b, Supporting Information). It further demonstrates the superiority of HMTA@MOF‐545‐Co that can combine HMTA and MOF‐545‐Co maximizes the electrocatalytic CO_2_RR performance. In this regard, we will select HMTA@MOF‐545‐Co with the optimal performance as the desired electrocatalyst for further electrocatalytic CO_2_RR investigation.

Besides, the *j*_CO_ is also a crucial factor to evaluate the catalytic performance of an electrocatalyst. Interestingly, the *j*_CO_ of HMTA@MOF‐545‐Co (20.6 mA cm^−2^ at −1.2 V) increases four times when compared with that of MOF‐545‐Co (5.1 mA cm^−2^ at −1.0 V) (Figure 2d), which implies that the introduction of HMTA possesses strong synergistic effect with MOF‐545‐Co. To further prove the effect of HMTA on the catalytic performance of MOF‐545‐Co, we have also prepared the physical mixture of MOF‐545‐Co and HMTA (HMTA/Co‐TCPP = 1:1) as the contrast sample and tested its electrocatalytic CO_2_RR performance (Figure 2f). It displays much lower FE_CO_ (70.8%, −0.8 V) and *j*_CO_ (7.9 mA cm^−2^ at −0.8 V) of physical mixture when compared with that of HMTA@MOF‐545‐Co (100.0%, −0.7 V; 11.2 mA cm^−2^ at −0.7 V), which demonstrates the superiority of the coordinated HMTA in the pore channels of MOF‐545‐Co that can boost the catalytic performance. Noteworthy, the highest FE_CO_ of HMTA@MOF‐545‐Co (100.0% at −0.7 V) can be almost twice higher than that of MOF‐545‐Co (56.9% at −0.9 V) (Figure 2f and Figure S14, Supporting Information). Furthermore, the electrochemical impedance spectroscopy (EIS) measurement is used to probe the electrocatalytic kinetics on the interface of electrode/electrolyte. Interestingly, the impedance value of HMTA@MOF‐545‐Co (3.35 Ω) almost doubly decreases in sharp contrast with that of MOF‐545‐Co (6.71 Ω), which indicates that HMTA in MOF‐545‐Co can largely facilitate the electron transfer from the catalyst to the reactants (Figure S15, Supporting Information). To further explore the potential influence factors for the excellent catalytic performance of HMTA@MOF‐545‐Co, the electrochemical double layer capacitance (*C*_dl_) is also calculated to estimate the electrochemical active surface area (ECSA) (Figure S16c, Supporting Information). Specifically, the *C*_dl_ of HMTA@MOF‐545‐Co and MOF‐545‐Co are 10.8 mF cm^−2^ and 17.2 mF cm^−2^, respectively. Similarly, the ECSA of HMTA@MOF‐545‐Co and MOF‐545‐Co is calculated to be 270 and 430 cm^2^, respectively, calculated based on the value of *C*_dl_ (Figure S16c, Supporting Information). The decreased ECSA may be attributed to the partially occupied active sites by the coordination of HMTA.^[^ ^35^, ^51^ ^]^ Furthermore, CO partial current density collected by ECSA of HMTA@MOF‐545‐Co is approximately eight times increased than that of MOF‐545‐Co (Figure S16d, Supporting Information), implying that the addition of HMTA can largely enhance the catalytic activity of MOF‐545‐Co in the electrocatalytic CO_2_RR process. Besides, the Tafel Slopes are also used to estimate the reaction kinetics for the CO generation. As shown in Figure S17 in the Supporting Information, the Tafel slope of HMTA@MOF‐545‐Co (180.4 mV dec^−1^) is much lower than that of MOF‐545‐Co (243.5 mV dec^−1^), indicating more favorable kinetics in electrocatalytic CO_2_RR.

To explore the stability of HMTA@MOF‐545‐Co, 10 h electrolysis is performed at −0.7 V versus reversible hydrogen electrode (RHE). During the electrolysis process, the electrolysis product is detected every 2 h through the GC measurement. During the process, FE_CO_ of HMTA@MOF‐545‐Co is stable at 99.95% after 10 h constant‐voltage electrolysis, and the current density remains constant (Figure S18a, Supporting Information). Meanwhile, the PXRD pattern shows that HMTA@MOF‐545‐Co can retain the origin structure after long‐term electrocatalysis reaction (Figure S18b, Supporting Information), indicating the high stability of HMTA@MOF‐545‐Co. To further explore the durability of the catalyst, EIS and ECSA have been detected after the stability experiment, the results showed that the samples still maintained the origin catalytic activity after 10 h catalytic reaction (Figures S19 and S20, Supporting Information).

**Figure 3:**
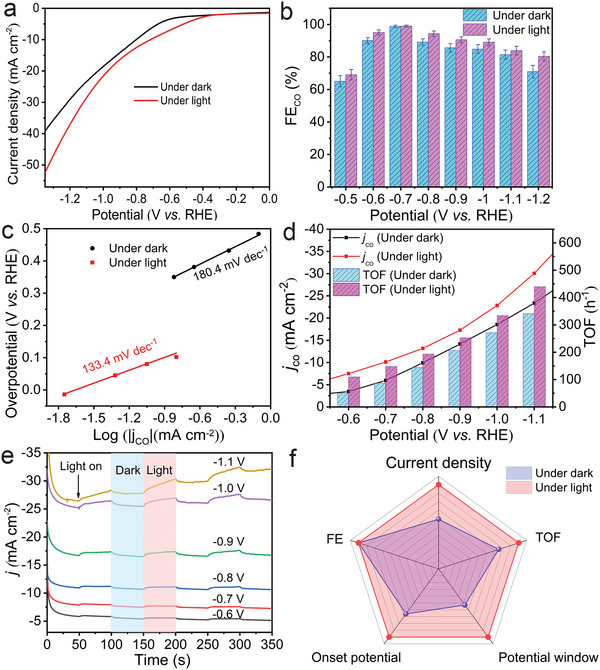
The electrocatalytic CO_2_RR performance of HMTA@MOF‐545‐Co under light and dark conditions. a) LSV curves of HMTA@MOF‐545‐Co. b) FE_CO_ of HMTA@MOF‐545‐Co. c) Tafel of HMTA@MOF‐545‐Co. d) *j*_CO_ and TOF for HMTA@MOF‐545‐Co measured under dark and light condition. e) Photoresponse current curves of HMTA@MOF‐545‐Co at different potential in H‐cell. f) The radar chart of performance comparison of HMTA@MOF‐545‐Co.

Additionally, many previous reported works have illustrated that photoassisted electrocatalysis was more beneficial for CO_2_ conversion into various value‐added products in contrast to the traditional electrocatalysis techniques.^[^ ^52^ ^]^ In this work, the Co‐TCPP unit in HMTA@MOF‐545‐Co, as an excellent photo responsive ligand,^[^ ^53^ ^]^ is expected to further increase the charge transfer and improve the electrocatalytic performance under visible light irradiation.^[^ ^30^, ^32^ ^]^ Therefore, the electrocatalytic performance of HMTA@MOF‐545‐Co is evaluated under the condition of light illumination. First, the LSV curves are measured under dark and light conditions to investigate the influence of light illumination on the CO_2_ reduction polarization potential (**Figure** **3**a). The result indicates that HMTA@MOF‐545‐Co exhibits lower overpotential for CO_2_ reduction under light illumination condition. In addition, the FE measurement of HMTA@MOF‐545‐Co shows that the FE_CO_ can reach above 90% at −0.5 to −0.9 V versus RHE, which is largely improved when compared with that of under dark conditions (Figure 3b). Interestingly, a highest energy efficiency of ≈69.89% can be achieved at −0.6 V under light condition, which is higher than that under dark condition (≈65.9%, −0.6 V) (Figure S21, Supporting Information). Noteworthy, its optimized reaction rate for CO generation can reach up to ≈5.11 mol m^−2^ h^−1^ at −1.2 V under light condition (Table S3, Supporting Information), which is much higher than that under dark condition (≈3.67 mol m^−2^ h^−1^ at −1.2 V).

The catalytic kinetics of electrocatalysts are also evaluated with Tafel slopes measured under dark and light conditions. The calculated Tafel slope of HMTA@MOF‐545‐Co under light illumination (133.4 mV dec^−1^) is much lower than that under dark condition (180.4 mV dec^−1^) (Figure 3c), indicating light illumination is conducive for the improved charge transfer kinetics of electrocatalytic CO_2_RR. Furthermore, the turnoverfrequency (TOF) and *j*_CO_ at different potentials of HMTA@MOF‐545‐Co are measured under both dark and light conditions. The results indicate that both TOF and *j*_CO_ exhibit obvious enhancement under light irradiation in a wide potential range from −0.5 to −1.2 V (Figure 3d). Besides, the photoresponse current test shows that the current density is significantly increased under light condition (Figure 3e). Based on the above experimental results, a radar chart is made to compare the catalytic performance under illumination and dark conditions to better demonstrate its superiority (Figure 3f). Under illumination condition, HMTA@MOF‐545‐Co shows broader potential window, lower onset potential, higher TOF and current density when compared with the values under dark condition. According to the above experimental results, the following conclusions can be drawn: light irradiation might promote the kinetics of electron transfer of HMTA@MOF‐545‐Co, improve the current density in the catalytic process, and thus enhance the activity of electrocatalytic CO_2_RR.

**Figure 4:**
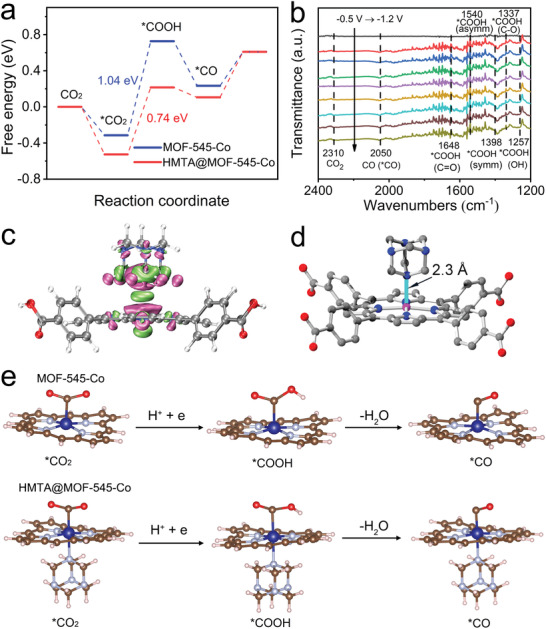
Mechanism and DFT calculations. a) Free energy profiles for CO_2_‐to‐CO reaction pathway on MOF‐545‐Co and HMTA@MOF‐545‐Co. b) In situ FTIR spectra of HMTA@MOF‐545‐Co for CO_2_RR. c) Charge interaction between HMTA and Co‐TCPP, green indicates an increase in charge density, and red indicates a decrease in charge density. d) The structural model of coordination between HMTA and Co‐TCPP. e) The CO_2_‐to‐CO conversion reactive pathway and intermediate architectures over MOF‐545‐Co and HMTA@MOF‐545‐Co.

During the CO_2_RR processes, the in situ attenuated total reflectance FTIR (ATR‐FTIR) studies are recorded to gain insight into the generation of intermediates, which could serve as vital evidence for further mechanism investigation. As displayed in **Figure** **4**b, the peaks detected at 1700–1200 cm^−1^ are assigned to the crucial intermediate (i.e., COOH*) for CO_2_RR to CO.^[^ ^54^ ^]^ Additionally, the 2050 cm^−1^ appeared is ascribed to the chemisorbed CO (*CO). The detected vital intermediates set solid basis for further theoretical study to investigate the related reaction mechanisms (Figure 4b).

To further certify the electrocatalytic CO_2_RR mechanism of HMTA@MOF‐545‐Co, the first principle^[^ ^54^, ^55^ ^]^ is used to perform the density functional theory(DFT) calculations within the generalized gradient approximation using the Perdew–Burke–Ernzerh^[^ ^56^ ^]^ formulation. Differential charge density distribution is firstly performed to simulate the electron structure, in which the green part shows the increase of charge density and the purple part indicates the decrease of charge density. The configurations of HMTA and Co‐TCPP are simulated and calculated by Orca software (Figure 4c). The results show that HMTA possesses strong interaction with the Co‐TCPP unit in HMTA@MOF‐545‐Co and can largely enhance the charge density of active sites. Furthermore, as shown in Figure 4d, the bond length between HMTA and Co‐TCPP (0.23 nm) is in the range of coordination bonds, indicating HMTA is linked with the Co atom of Co‐TCPP through a coordinate bond. In addition, based on our theoretical simulation model, we calculated the theoretical model of the intermediate on the catalyst for each step of the CO_2_RR process (Figure 4e). In general, the conversion process of CO_2_‐to‐CO includes the adsorption/activation of CO_2_ to generate *CO_2_, transformation into *COOH and then *CO, and finally CO desorption.^[^ ^51^ ^]^ In the case of MOF‐545‐Co (Figure 4a), the interaction force between CO_2_ molecule and Co‐TCPP is rather weak, and the binding energy is only −0.31 eV (Figure 4a). The Gibbs free energy from the adsorbed *CO_2_ to *COOH is calculated to be 1.04 eV, which is also the determining step for the formation of CO. Then, the *COOH transforms into *CO and finally need 0.49 eV for the CO desorption on MOF‐545‐Co. For HMTA@MOF‐545‐Co, the modification of HMTA can largely increase the CO_2_ adsorption energy to −0.53 eV, which is almost doubly enhanced (Figure 4a). Noteworthy, its Gibbs free energy for the rate determining step from *CO_2_ to *COOH largely decreases to 0.74 eV when compared with that of MOF‐545‐Co, which matches well with the above‐mentioned experimental results. After that, only −0.14 eV energy is needed for the transformation of *COOH into *CO. Finally, *CO undergoes the desorption process to generate CO. Thus, the introduction of HMTA generates strong coordination bond with MOF‐545‐Co that can increase the charge density of active sites, almost doubly enhance the CO_2_ adsorption energy and largely reduce the energy barrier of rate determining step to boost the CO_2_RR performance. This may be due to the relatively long electron transfer distance at the N site during the electrocatalytic process.

## 3 Conclusion

In summary, we report an oriented coordination strategy to introduce N‐rich auxiliary (i.e., HMTA) into metalloporphyrin metal–organic frameworks to synthesize a series of site‐specific functionalized electrocatalysts (HMTA@MOF‐545‐M, M = Fe, Co, and Ni) and successfully apply them in light‐assisted CO_2_ electroreduction. After modification, HMTA can be coordinated with the metalloporphyrin centers in the pore channels of MOF‐545‐M (M = Fe, Co, and Ni), inducing strong interaction with CO_2_ molecules. Specifically, thus‐obtained HMTA@MOF‐545‐Co presents approximately two times enhanced CO_2_ adsorption‐enthalpy and electrochemical active surface‐area with largely decreased impedance‐value after modification, resulting in almost twice higher CO_2_ electroreduction performance than its unmodified counterpart. Noteworthy, HMTA@MOF‐545‐Co displays superior FE_CO_ (≈100%) in a wide potential range −0.7 to −1.1 V versus RHE, a high CO generation rate (≈5.11 mol m^−2^ h^−1^ at −1.1 V) and high energy efficiency (≈70% at −0.7 V) under light irradiation, which is superior to most of state‐of‐the‐art electrocatalysts. and its catalytic performance under dark conditions. As certified by the theoretical calculations, the oriented coordination of HMTA can facilitate the CO_2_ adsorption/activation, increase the charge density of active sites and largely reduce the rate‐determining step for the boosted performance improvement. We anticipate that our strategy, combined with the rich chemistry of MOFs and modifiable N‐rich agents, may promote the development of functional porous crystalline materials for highly efficient electrocatalytic CO_2_RR.

## 4 Experimental Section

### Synthesis of MOF‐545

ZrOCl_2_·8H_2_O (650 mg, 2.0 mmol) and tetrakis (4‐carboxyphenyl)porphyrin (TCPP‐H_2_, 130 mg, 0.192 mmol) were dissolved in 160 mL N,N'‐dimethylformamide (DMF) in a 250 mL round‐bottomed flask and 5 mL dichloroacetic acid is added to the above solution. The reaction system was heated at 130 °C for 15 h with continuous stirring. After cooling to room temperature, the solid was collected by centrifugation, and washed with DMF until the liquid become colorless. Then, the resulting powder was dispersed in a mixed solution containing 50 mL DMF and 5 mL 1 m HCl, and refluxed for 2 h. After centrifugation, the solid was washed with DMF and acetone for several times and then soaked in acetone overnight. The powder was washed with acetone and dried at 60 °C under vacuum. After drying, about 200 mg purple powder was collected (yield 85% based on TCPP‐H_2_).

### Syntheses of MOF‐545‐M (M = Fe, Co, Ni)

MOF‐545 (50 mg) was dispersed in 10 mL DMF solution of CoCl_2_·6H_2_O (100 mg, 0.42 mmol). After sonicating for 10 min, the solution was heated at 100 °C for 20 h. After heating, purple powder was collected by centrifugation and washed with DMF for several times until the liquid become colorless. The DMF was replaced with acetone (5 × 30 mL) over a 3 d period. Finally, the powder was dried in vacuum at 60 °C overnight. The preparation processes of MOF‐545‐Fe and MOF‐545‐Ni were similar to that of MOF‐545‐Co except that CoCl_2_·6H_2_O was replaced by FeCl_3_·6H_2_O (100 mg) and NiCl_2_·6H_2_O (100 mg), respectively.

### Synthesis of HMTA@MOF‐545‐Co

After degassing at 120 °C for 8 h under dynamic vacuum, the activated MOF‐545‐Co (50 mg) was added in a 50 mL Schlenk tube. 1 mL of HMTA (134 mg) in CHCl_3_ (1 mL) solution was dropped on the powder until the solution just immersed the MOF‐545‐M powder. Subsequently, the Schlenk tube was tighten and sonicated for 1 h. The powder was collected and washed with CHCl_3_ for five times to completely wash off the unsupported HMTA. After drying under vacuum at 60 °C for 8 h, about 56 mg product was obtained. HMTA@MOF‐545‐Fe and HMTA@MOF‐545‐Ni were prepared by the similar method as that of HMTA@MOF‐545‐Co except that MOF‐545‐Co was replaced by MOF‐545‐Fe or MOF‐545‐Ni, respectively. For the preparation of HMTA@MOF‐545‐Co with different HMTA loading amounts, another two amounts of HMTA (i.e., 33.5 mg and 67 mg) were applied to replace 134 mg HMTA and synthesized under similar procedures. Thus, HMTA@MOF‐545‐Co with three kinds of HMTA loadings were prepared for further characterizations.

## Conflict of Interest

The authors declare no conflict of interest.

## Author Contributions

Z.X., Y.C., and Y.‐Q.L. conceived and designed the idea. X.D., Y.‐R.W., Q.W. designed the experiments, collected and analyzed the data. K.S. and J.‐W.S. assisted with the experiments and characterizations. Z.X. wrote the paper. All authors have approved the final version of the paper.

## Supporting information

Supporting Information

## Data Availability

The data that support the findings of this study are available in the supplementary material of this article.
