# Supplementary material for: Electronic Tuning of CO2 Interaction by Oriented Coordination of N‐Rich Auxiliary in Porphyrin Metal–Organic Frameworks for Light‐Assisted CO2 Electroreduction
Source: Adv Sci (Weinh). 2023 May 1;10(21):2301261. doi: 10.1002/advs.202301261 (PMC10375083; doi:10.1002/advs.202301261)
Supplement: Supplementary file 1 — Supporting Information [file ADVS-10-2301261-s001.pdf]

ADVANCED SCIENCE
Open Access

Supporting Information

for *Adv. Sci.*, DOI 10.1002/advs.202301261

Electronic Tuning of CO_2 Interaction by Oriented Coordination of N-Rich Auxiliary in Porphyrin Metal–Organic Frameworks for Light-Assisted CO_2 Electroreduction

Zhifeng Xin, Xue Dong, Yi-Rong Wang, Qian Wang, Kejing Shen, Jing-Wen Shi, Yifa Chen* and Ya-Qian Lan*

Supporting Information

Electronic Tuning of CO_2 Interaction by Oriented Coordination of N-Rich Auxillary in Porphyrin Metal-Organic Frameworks for Light-assisted CO_2 Electroreduction

Zhifeng Xin[1], Xue Dong[1], Yi-Rong Wang[2], Qian Wang[1], Kejing Shen[1], Jing-Wen Shi[2], Yifa Chen[2]*, Ya-Qian Lan[2]**

[1]Z. X., X. D., Q. W., and K. S.

Institute of Molecular Engineering and Applied Chemistry

Anhui University of Technology

Ma'anshan, Anhui 243002, P. R. China

E-mail: xinzf521@ahut.edu.cn

[2]Y.-R. W., J.-S. S., Prof. Y. C., and Prof. Y.-Q. L.

School of Chemistry

South China Normal University

Guangzhou, 510006, P. R. China

E-mail: chyf927821@163.com

Experimental Procedures

Materials

All chemicals and solvents were commercially available and used without further purification. Zirconyl chloride octahydrate ($ZrOCl_2 \cdot 8H_2O$), N,N'-dimethylformamide (DMF), acetone, ferrous chloride ($FeCl_3 \cdot 6H_2O$), nickel chloride ($NiCl_2 \cdot 6H_2O$) and cobalt chloride ($CoCl_2 \cdot 6H_2O$) are purchased from Sinopharm Chemical Reagent Co., Ltd. Meso-tetra(4-carboxyphenyl) porphyrin and $CHCl_3$ are purchased from Shanghai Taitan Scientific Co., Ltd. All aqueous solutions are prepared with Millipore water (18.25 MΩ).

Characterizations and instruments

Powder X-ray diffraction (PXRD) experiments are recorded on Bruker D8 Advance (operating at 40 kV and 20 mA) with Ni-filtered Cu Ka radiation at 1.5406 (Å) with a speed of 5 ^{o} min^{-1}. SEM images is obtained from a FEI NOVA NANO 430 Field Emission Scanning Electron Microscope equipped with an Oxford Energy Dispersive X-ray spectroscopy. N_2 and CO_2 sorption measurements are carried out on a Micromeritics ASAP 2460 system at 77 K and 298 K, respectively, after the samples are first degassed at 120 ^{o}C for 8 h. Raman spectra are collected on a Renishaw in Via Raman Spectrometer. Fourier transform infrared (FTIR) spectra are acquired from a Nicolet 6700 spectrometer (Nicolet Instrument Co., USA). The thermo-gravimetric analysis (TGA) was performed on a Shimadzu DTG-60H thermo-gravimetric analyzer in N_2 flow of 30 mL min^{-1} and the heating rate of 10 ^{o}C min^{-1}. X-ray Photoelectron Spectroscopy (XPS) was carried out on a Thermo ESCALAB 250XI multifunctional imaging electron spectrometer using the binding energy of C as the internal standard. TEM images and STEM-HAADF images coupled to EDS elemental mapping were collected on a JEOL JEM-2100 electron microscope at 200 kV equipped with an Oxford Energy Dispersive X-ray spectroscopy.

The preparation of working electrode

10 mg sample and 10 mg acetylene black were grinded for 10 min and dispersed in 1 mL 0.5% Nafion solution followed with sonication for 30 min to form uniform catalyst ink. The ink was dropped directly on a hydrophobic carbon paper (1 cm × 2 cm) to form a 1 × 1 cm^2 catalyst area with a catalyst loading of ~1 mg cm^{-2} (cathode) and ~2 mg cm^{-2} (anode). The deposited carbon paper was further dried at room temperature.

Electrolysis and analysis of CO_2 reduction product

All electrochemical tests are performed in a standard three-electrode configuration in 0.5 M $KHCO_3$ solution using a CHI660-E electrochemical workstation. Carbon rod and Ag/AgCl are used as counter electrode and reference electrode, respectively, and modified carbon paper (1 cm ×1 cm) is used as work electrode. The electro-chemical CO_2RR performance is carried out in an airtight electro-chemical H-type cell, in which, two compartments are separated by a Nafion®117 proton exchange membrane to prevent mixing of products from the two electrode chambers. The polarization curves are performed by linear sweep voltammetry (LSV) mode at a scan rate of 5 mV s^{-1}. In this experiment, polarization curves are recorded successively in Ar-saturated and CO_2 saturated $KHCO_3$ solution. The measurement of electrochemical impedance spectroscopy (EIS) is carried out under the overpotential of -0.7 V (relative to RHE). During the measurement process, 10 mV amplitude AC voltage is applied in the frequency range of 1000 kHz to 100 MHz. To estimate the electrochemical active surface area (ECSA), cyclic voltammograms (CV) are tested under the potential window of -0.1 V – 0 V (vs. Ag/AgCl) with various scan rates from 20 to 100 mV s^{-1}. In this work, all the potentials are measured vs. Ag/AgCl electrode and the results are calculated to the potential vs. reversible hydrogen electrode (RHE) based on the Nernst equation: E (vs. RHE) = E (vs. Ag/ AgCl) + 0.1989 V +0.059 × pH (without iR compensation).

The gaseous reduction products were monitored by a gas chromatography (Shimadzu-2010 plus) equipped with a flame ionization detector (FID) and a thermal conductivity detector (TCD). The liquid

products (e.g. formate) were collected from the anode chambers after electrolysis and quantified by NMR. In this work, all reported values are average ones calculated from three or more independent measurements, and all errors are given as standard deviations.

Evaluation of CO_2RR performance

For products, the Faradaic efficiency (FE_{CO}) was calculated according to the following equation:

$$FE_{CO} = \frac{N \times F \times n_{products}}{Q} \times 100\%$$

N: the number of electrons transferred for product formation (N = 2 for CO).

F: Faraday constant, 96485 C mol^{-1};

$n_{products}$: the moles of products;

Q: the total charge obtained from chronoamperometry (C).

The turnover frequency (TOF, h^{-1}) of CO was calculated by the equation:

$$TOF\ (h^{-1}) = \frac{i_{total} \times FE_{CO}}{N \times F \times n_{cat.}}$$

i_{total}: the total current (A);

FE_{CO}: the faraday efficiency of CO (%);

N: the number of electrons in the half reaction (N = 2 for CO_2 to CO conversion);

F: the Faraday constant (96485 C/mol);

$n_{cat.}$: the moles of catalyst employed in the electrolysis (mol).

The calculation of energy efficiency (EE, %)

$$EE\ (\%) = \frac{E_j^0 \times FE_j}{E_j^0 + \eta} \times 100\%$$

E_j^0: the equilibrium cell potential for a certain product (V).;

FE_j: the Faradaic efficiency of the aiming product j (%);

η : the overpotential (V).

Reaction rate of the catalysts (r, mol h^{-1} m^{-2}):

$$r = \frac{j \times FE}{n \times F}$$

j: total current density;

n: the number of transferred electrons;

F: Faradaic constant (96485 C mol^{-1}).

Computational details

The first principle was used to perform all density functional theory (DFT) calculations within the generalized gradient approximation (GGA) using the Perdew-Burke-Ernzerh of (PBE) formulation. The projected augmented wave (PAW) potentials were chosen to describe the ionic cores and take valence electrons into account using a plane wave basis set with a kinetic energy cutoff of 400 eV. Partial

occupancies of the Kohn−Sham orbitals were allowed using the Gaussian smearing method and a width of 0.05 eV. The electronic energy was considered self-consistent when the energy change was smaller than 10−4 eV. A geometry optimization was considered convergent when the energy change was smaller than 0.04 eV $Å^{-1}$. In our structure, the U correction is used for Co atoms. The Brillouin zone integration is performed using 2×2×1 Monkhorst-Pack k-point sampling for a structure. Finally, the adsorption energies (Eads) were calculated as Eads= Ead/sub -Ead -Esub, where Ead/sub, Ead, and Esub are the total energies of the optimized adsorbate/substrate system, the adsorbate in the structure, and the clean substrate, respectively. The free energy was calculated using the equation:

G=Eads+ZPE-TS

where G, Eads, ZPE and TS are the free energy, total energy from DFT calculations, zero point energy and entropic contributions, respectively.

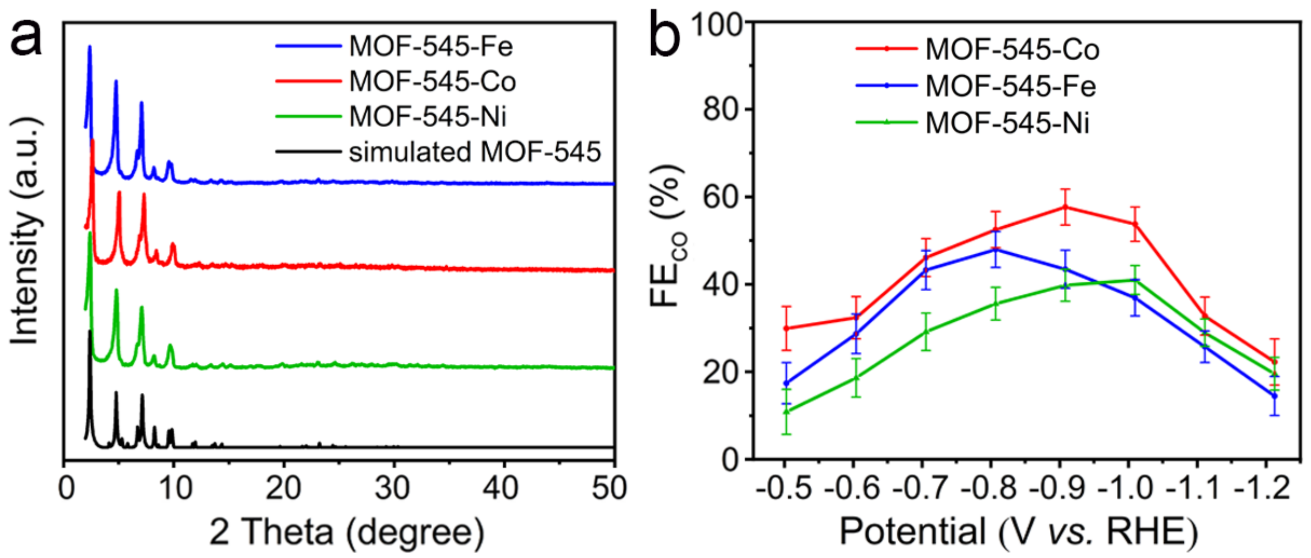

Figure S1. PXRD patterns and FE_{CO} of MOF-545-Co, MOF-545-Fe and MOF-545-Ni. (a) PXRD patterns. (b) FE_{CO}.

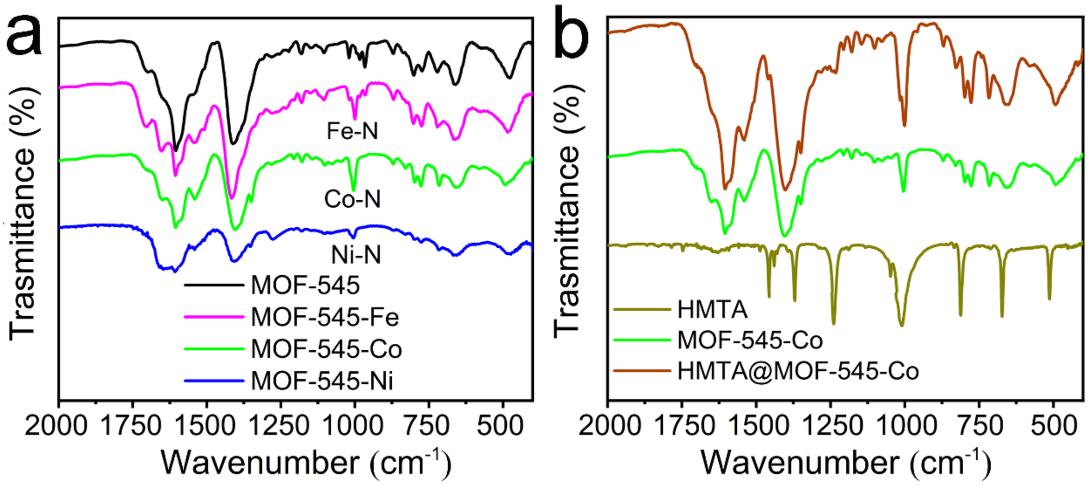

Figure S2. FT-IR spectra of samples. (a) MOF-545 and M-Loaded MOF-545 (M = Fe, Co and Ni). (b) HMTA, MOF-545-Co and HMTA@MOF-545-Co.

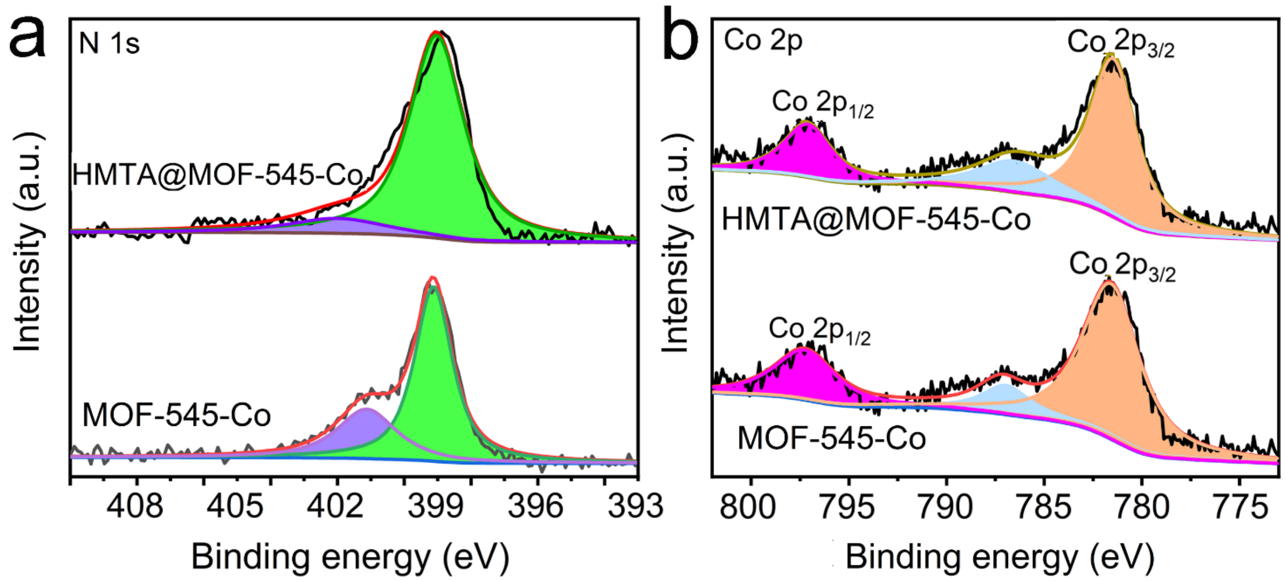

Figure S3. XPS spectra of MOF-545-Co and HMTA@MOF-545-Co. (a) XPS high-resolution scan of N 1s. (b) XPS high-resolution scan of Co 2p.

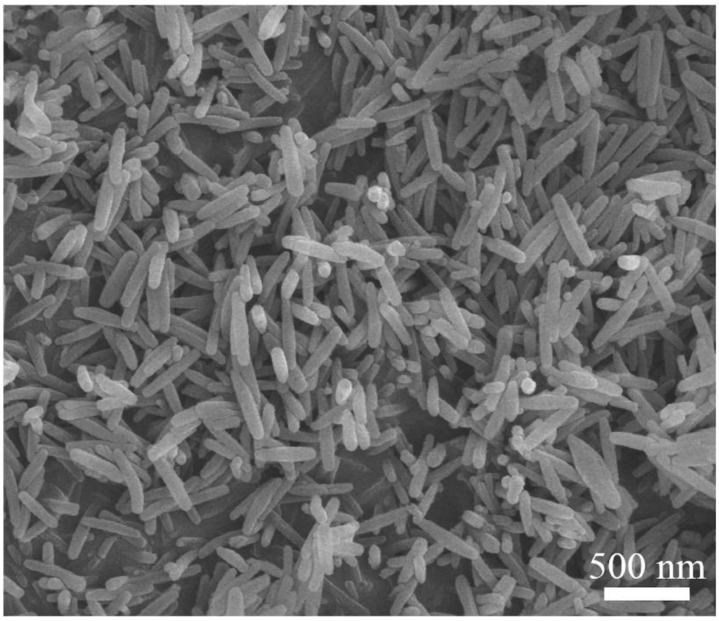

Figure S4. SEM image of MOF-545-Co.

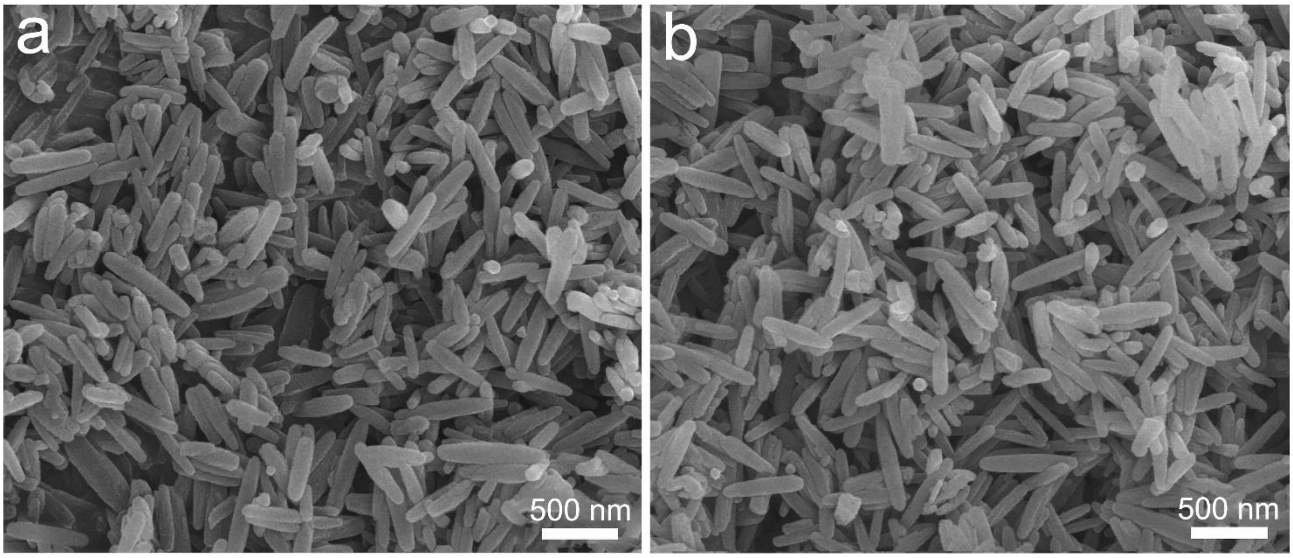

Figure S5. SEM image of HMTA@MOF-545-Fe and HMTA@MOF-545-Ni. a) HMTA@MOF-545-Fe. b) HMTA@MOF-545-Ni.

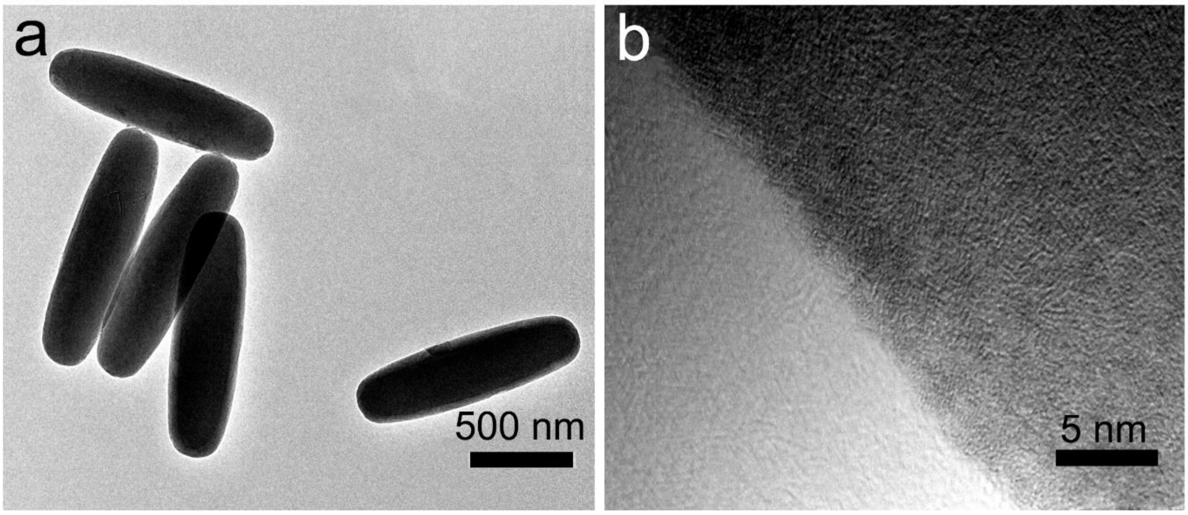

Figure S6. TEM images of of HMTA@MOF-545-Co. a) TEM image of of HMTA@MOF-545-Co. b) HRTEM image of HMTA@MOF-545-Co.

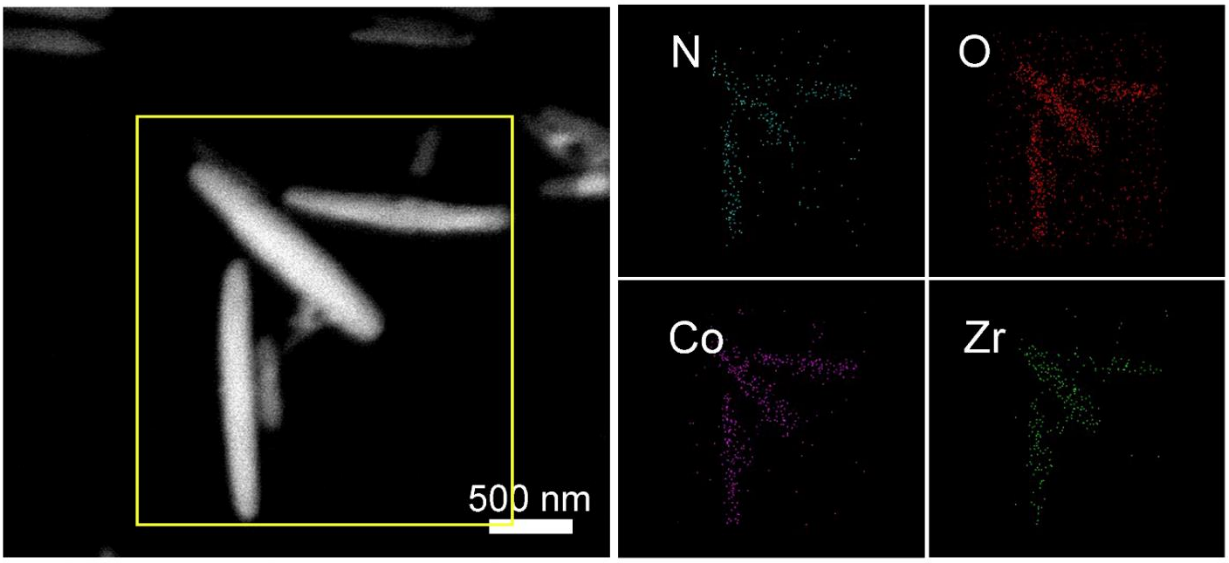

Figure S7. STEM-HAADF image and EDS elemental mapping images of HMTA@MOF-545-Co.

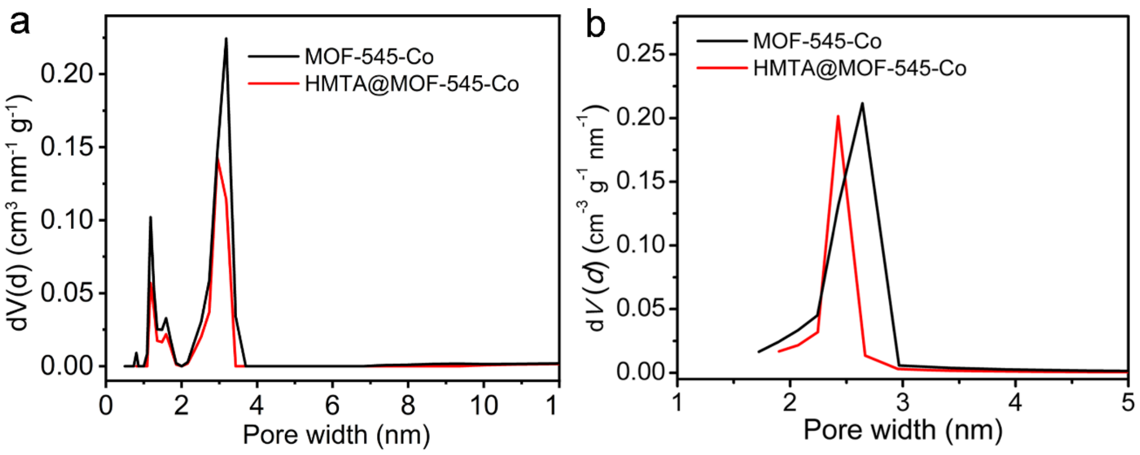

Figure S8. Calculated pore size distribution of HMTA@MOF-545-Co and MOF-545-Co. a) DFT result from N_2 adsorption data. b) BJH result.

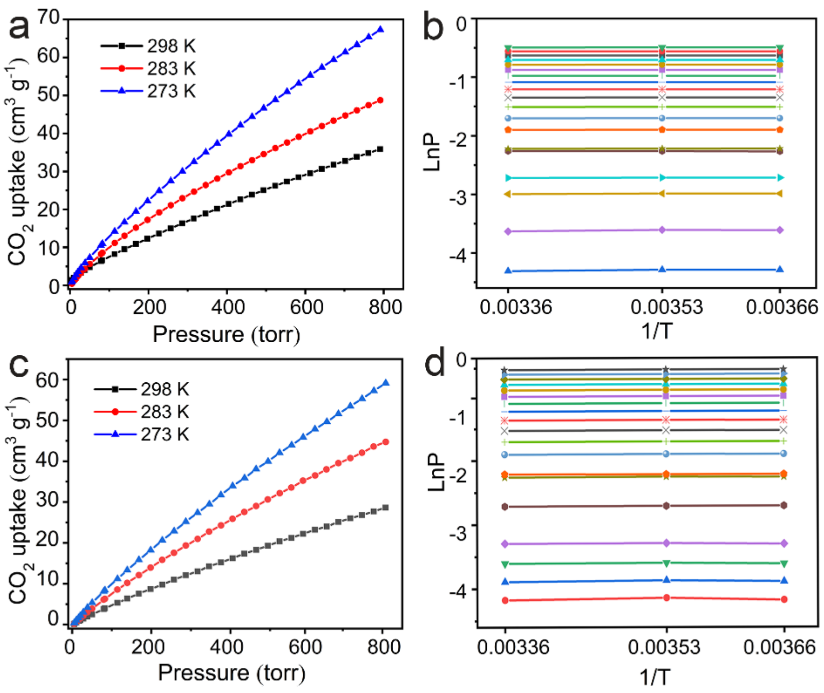

Figure S9. CO_2 adsorption performances. (a) CO_2 adsorption curves of MOF-545-Co at different temperatures. (b) CO_2 adsorption isosteres of MOF-545-Co. (c) CO_2 adsorption curves of HMTA@MOF-545-Co at different temperatures. (d) CO_2 adsorption isosteres of HMTA@MOF-545-Co.

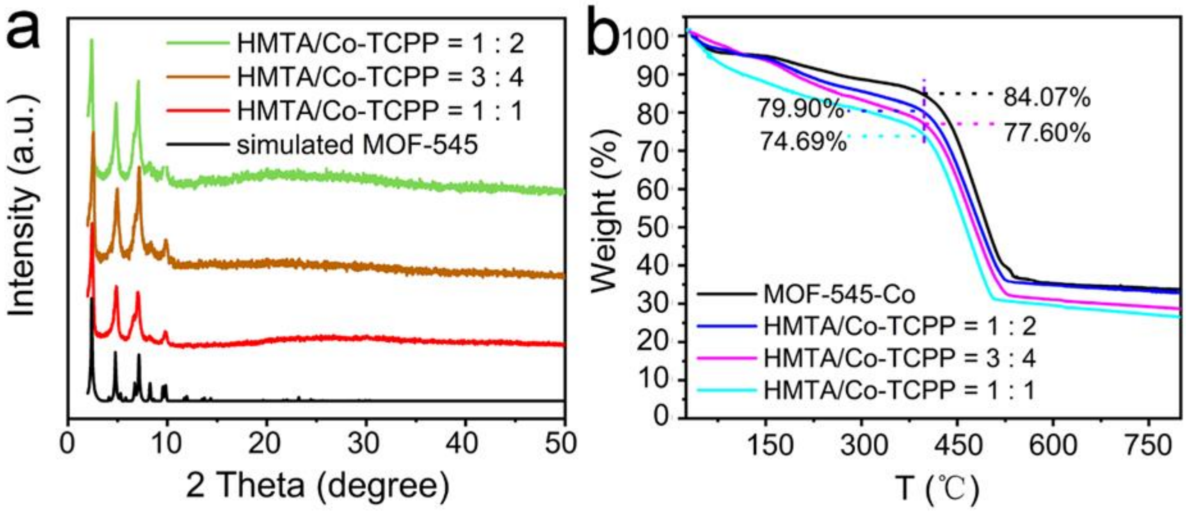

Figure S10. PXRD patterns and TGA curves of HMTA@MOF-545-Co with different HMTA loadings. (a) PXRD patterns. (b) TGA curves.

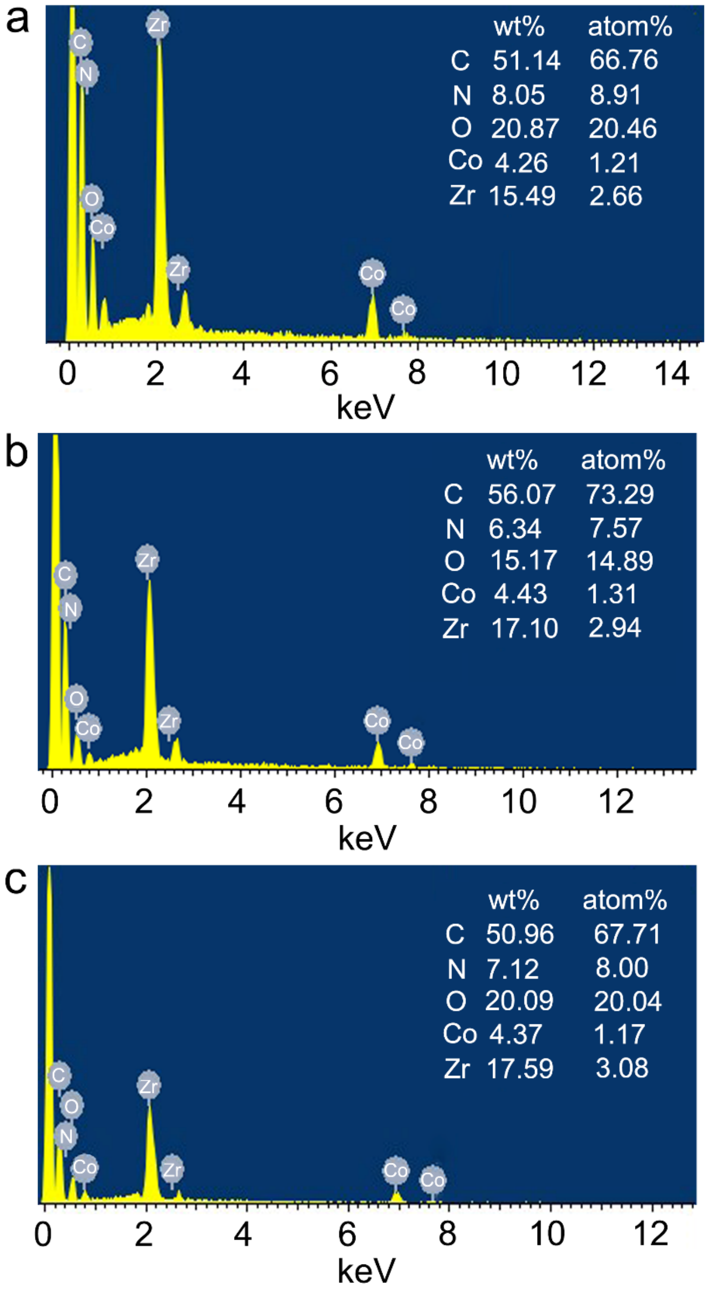

Figure S11. EDS images of HMTA@MOF-545-Co with different HMTA loadings. (a) HMTA/Co-TCPP = 1 : 1. (b) HMTA/Co-TCPP = 1 : 2. (c) HMTA/Co-TCPP = 3 : 4.

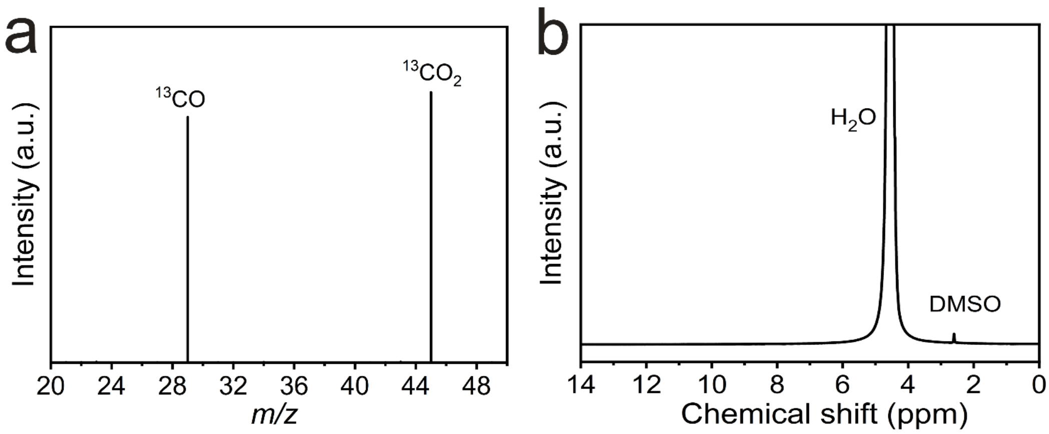

Figure S12. The mass and NMR spectra after 2 h CO_2RR process at -0.7 V catalyzed by HMTA@MOF-545-Co. (a) gas product of ^{13}CO recorded under $^{13}CO_2$ atmosphere. (b) ^{1}H NMR spectroscopy of the liquid product.

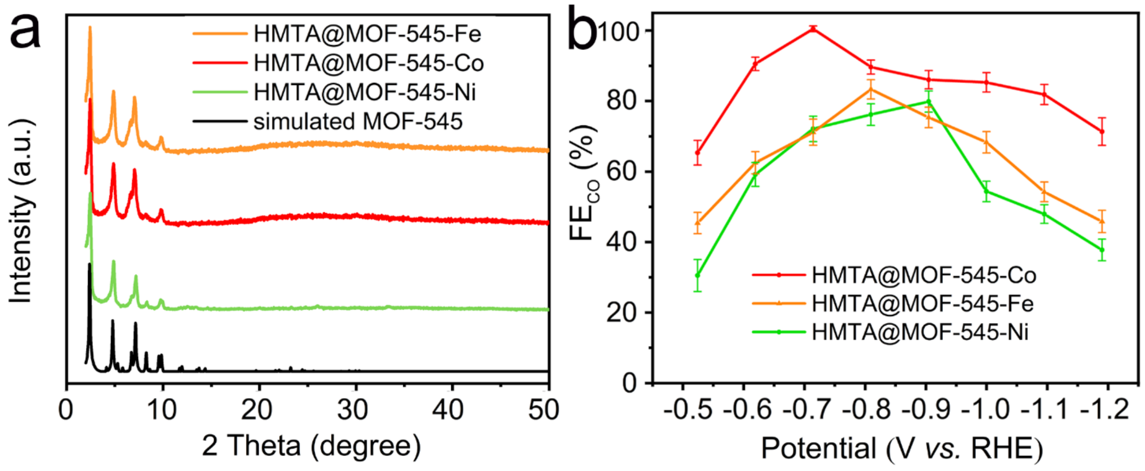

Figure S13. PXRD patterns and FE_{CO} of HMTA@MOF-545-Co, HMTA@MOF-545-Fe and HMTA@MOF-545-Ni. (a) PXRD patterns. (b) FE_{CO}.

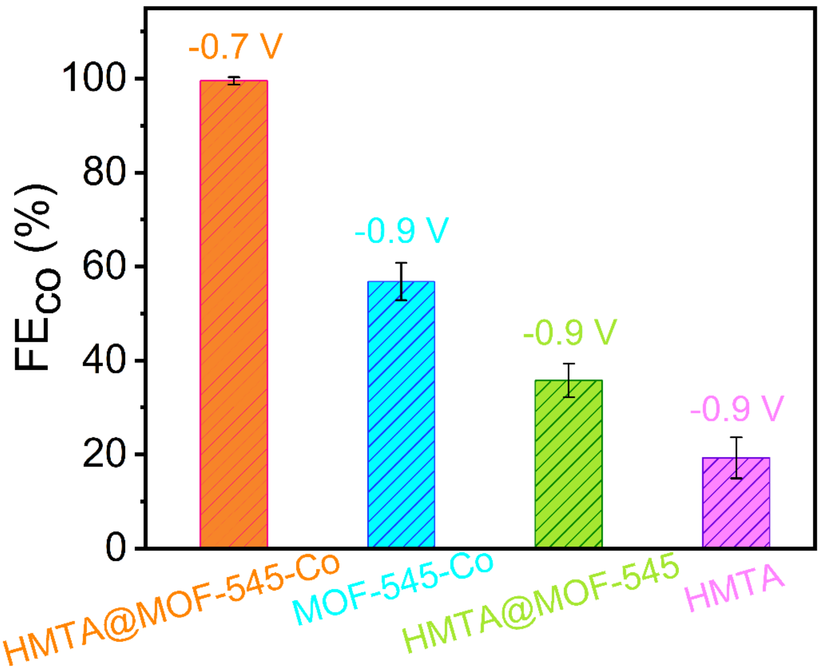

Figure S14. The maximum FE_{CO} of HMTA, HMTA@MOF-545, MOF-545-Co and HMTA@MOF-545-Co.

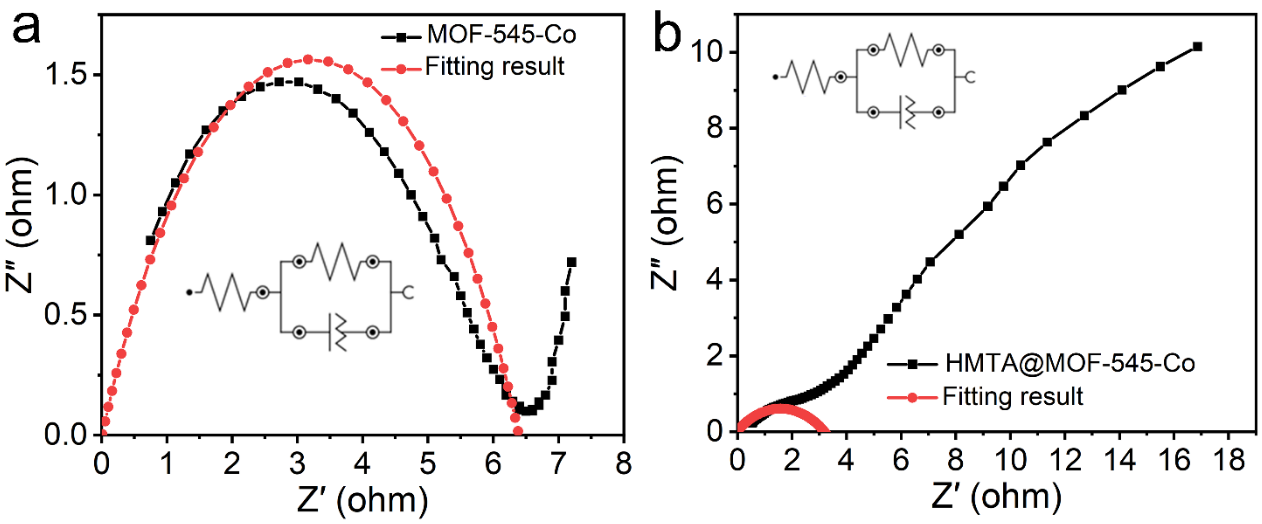

Figure S15. Nyquist plots of MOF-545-Co and HMTA@MOF-545-Co. (a) Nyquist plots and fitting result of MOF-545-Co. (b) Nyquist plots and fitting result of HMTA@MOF-545-Co.

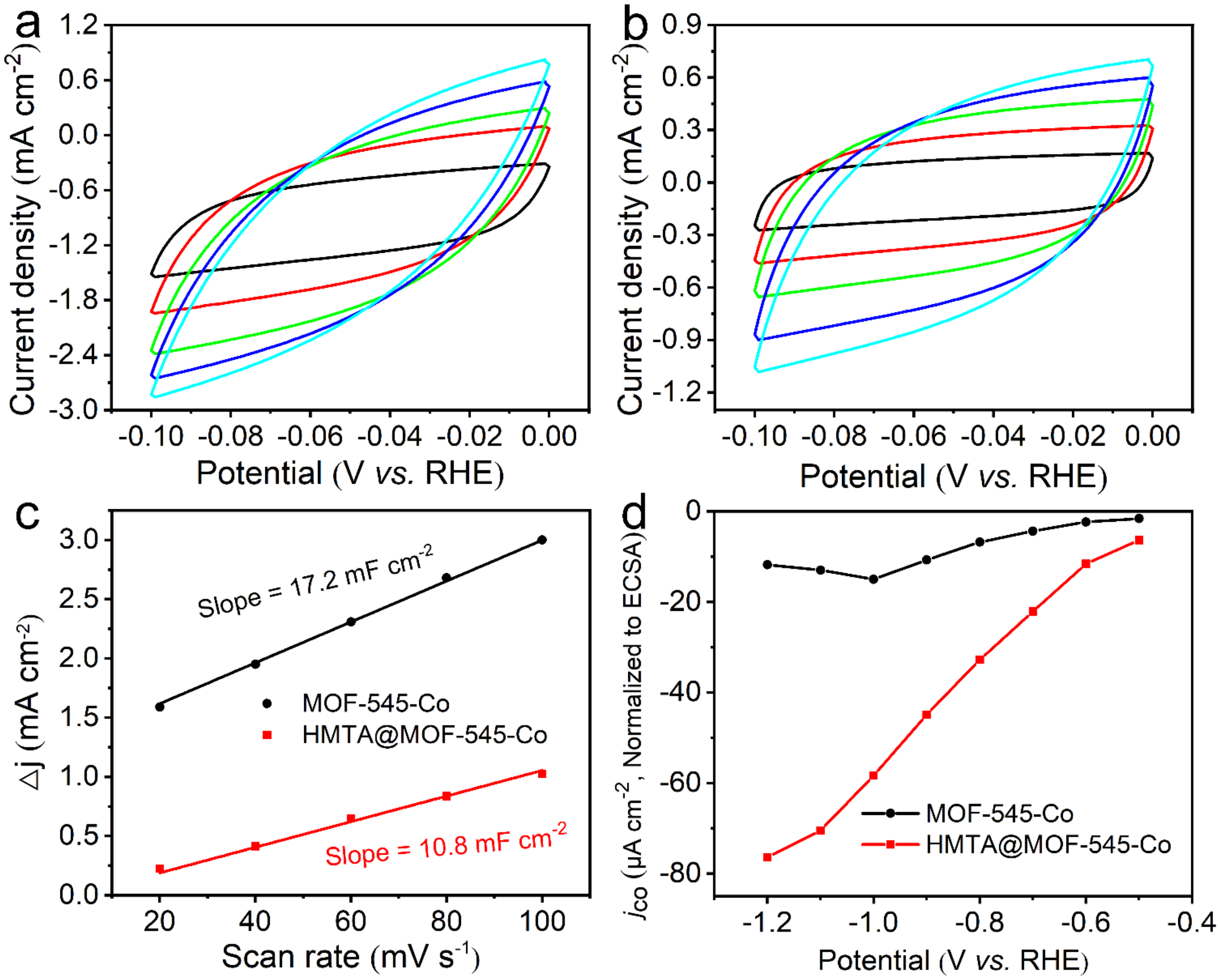

Figure S16. Electrochemical properties of MOF-545-Co and HMTA@MOF-545-Co. (a) CV curves of MOF-545-Co at different scanning rate (20-100 mV S^{-1}). (b) CV curves of HMTA@MOF-545-Co at different scanning rate (20-100 mV S^{-1}). (c) C_{dl}. (d) ECSA-normalized CO partial current density.

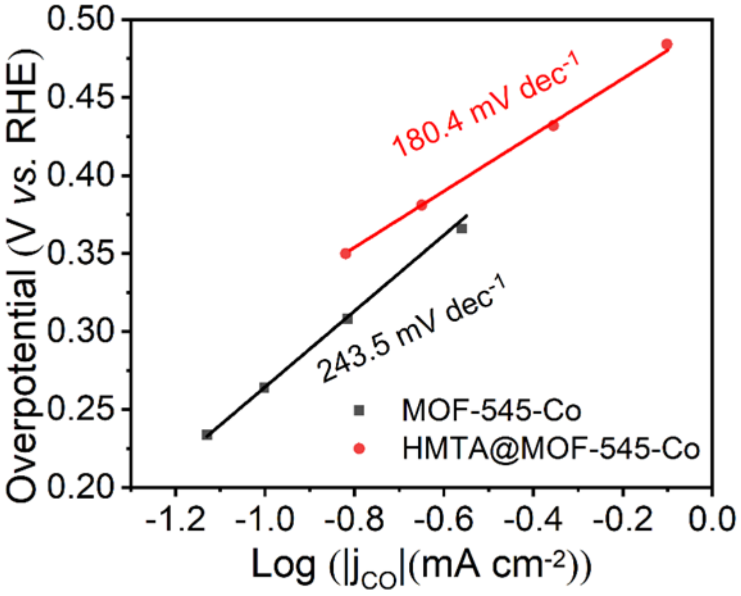

Figure S17. Tafel slopes of MOF-545-Co and HMTA@MOF-545-Co.

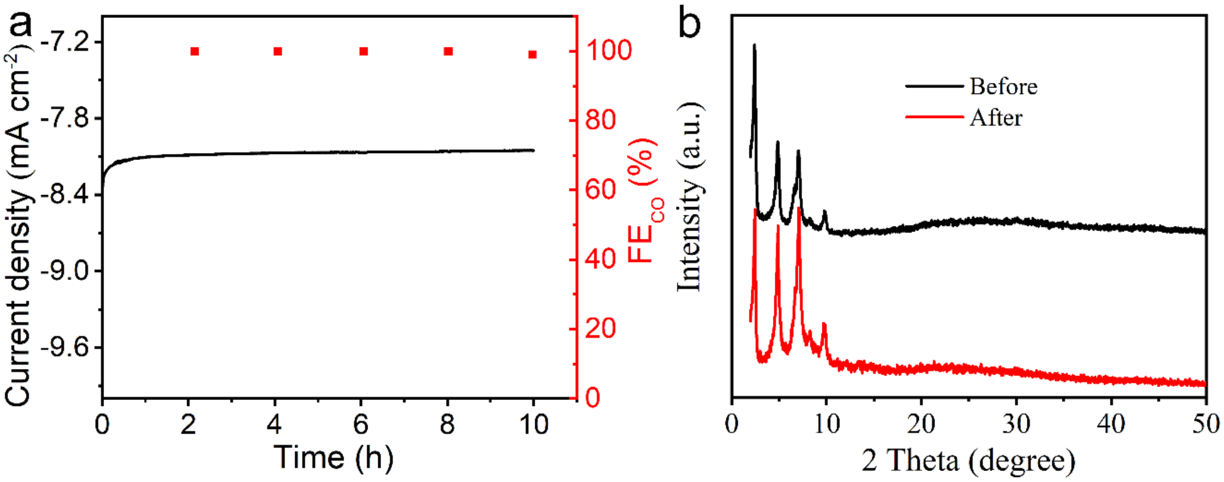

Figure S18. Stability test for HMTA@MOF-545-Co. (a) Long-term stability for CO_2RR at -0.7 V. (b) PXRD pattern of before and after electrocatalytic reactions.

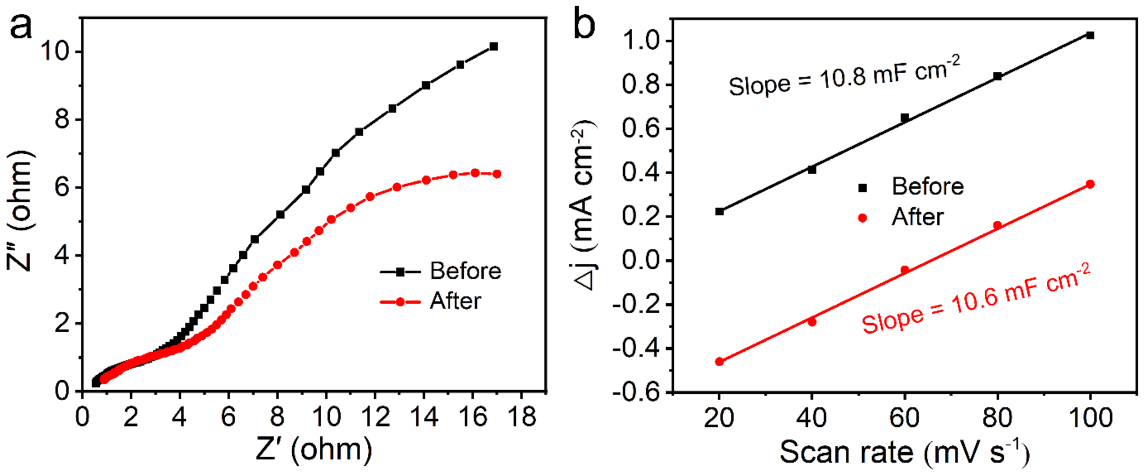

Figure S19. Electrochemical properties of HMTA@MOF-545-Co before and after stability test. (a) Nyquist plots. (b) C_{dl}.

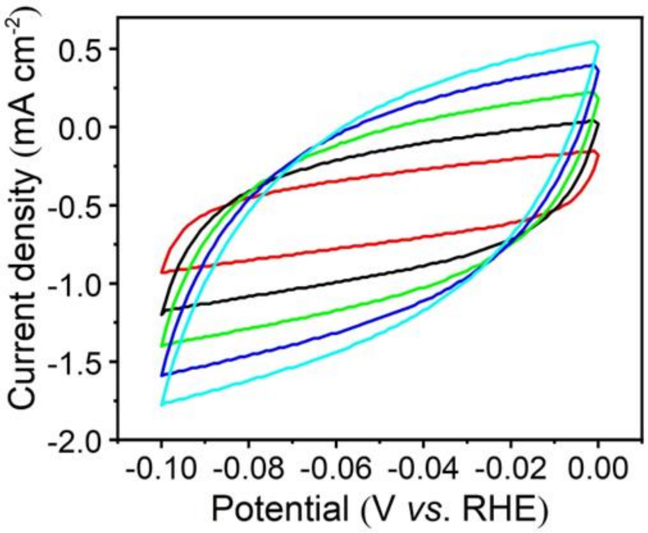

Figure S20. CV curves of HMTA@MOF-545-Co after electrochemical stability test measured at different scan rates (20-100 mV S^{-1}).

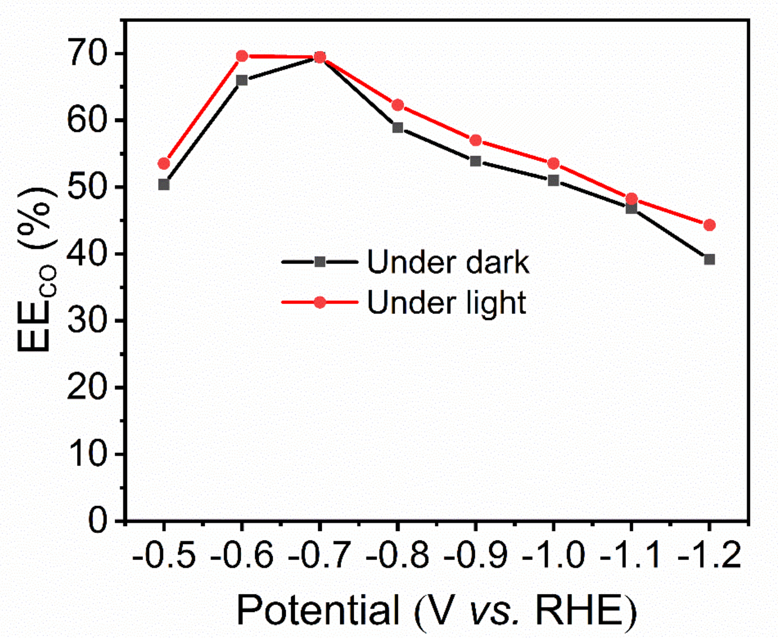

Figure S21. Energy efficiency (EE) of CO for HMTA@MOF-545-Co at dark and light conditions.

Table S1. The BET surface areas and pore volumes of HMTA@MOF-545-Co, MOF-545-Co, HMTA@MOF-545 and MOF-545 calculated through the N_2 adsorption tests.

**Samples**	**S_{BET} (m^2 g^{-1})**	**V_t (m^3 g^{-1})**	**V_{micro} (m^3 g^{-1})**	**$V_{meso/macro}$ (m^3 g^{-1})**
HMTA@MOF-545-Co	1316	0.79	0.44	0.35
MOF-545-Co	1957	1.38	0.73	0.65
HMTA@MOF-545	1730	1.27	0.68	0.59
MOF-545	2023	1.51	0.83	0.68

Table S2. The elemental/ICP analyses, and EDS results of HMTA@MOF-545-Co with different HMTA loadings.

	**Elemental analysis (wt%)**			**EDS (wt%)**		
C	50.24	55.97	50.79	51.14	56.07	50.96
N	7.95	6.14	7.16	8.05	6.34	7.12
O	20.47	14.98	19.79	20.87	15.17	20.09
Co	4.56	4.45	4.47	4.26	4.43	4.37
Zr	14.99	17.18	17.64	15.49	17.10	17.59
HMTA/Co-TCPP	1:1	1:2	3:4	1:1	1:2	3:4

Table S3. The summary of CO_2 electroreduction performances for reported porphyrin-based MOFs.

	Catalyst	FE_{CO} (%)	j_{CO} (mA cm^{-2})	Potential (V *vs.* RHE)	r (mol m^{-2} h^{-1})	Ref.
**1**	HMTA@MOF-545-Co-light	~100.0%	10.10	-0.7	5.11 (-1.20 V)	This work
**2**	HMTA@MOF-545-Co	99.6%	6.04	-0.7	3.76 (-1.20 V)	This work
**3**	MOF-545-Co	56.9%	5.1	-0.9	1.21 (-1.20 V)	This work
**4**	HMTA@MOF-545	36.4%	2.3	-0.9	0.54 (-1.20 V)	This work
**5**	Fe-MOF-525	60.0%	2.3	-1.3	0.4	[1]
**6**	[Al(OH)$_2$TCPP-Co]MOF	76%	1.0	-0.7	0.43 (-0.9 V)	[2]
**7**	PCN-222-Fe	91.0%	1.2	-0.6	0.75 (-0.85V)	[3]
**8**	PCN-222-Cu	44%	3.2	-0.7	1.78 (-0.90 V)	[4]
**9**	PCN-224-Cu	34%	2.4	-0.7	1.26 (-0.9 V)	[4]
**10**	CoCp$_2$@MOF-545-Co	97.0%	15.6	-0.7	4.78 (-0.9 V)	[5]
**11**	FeCp$_2$@MOF-545-Co	94.1%	14.9	-0.8	2.7	[5]
**12**	Fe-PMOF	29.0%	4.9	-0.7	0.47 (-1.10 V)	[6]
**13**	Co-PMOF	99.0%	19.5	-0.8	5.2 (-1.10 V)	[6]
**14**	Ni-PMOF	19.0%	3.2	-0.8	0.30 (-1.10 V)	[6]

References

[1] I. Hod, S M. D. ampson, P. Deria, C. P. Kubiak, O. K. Farha, J. T. Hupp, *ACS Catal.*, **2015**, *5*, 6302-6309

[2] N. Kornienko, Y. Zhao, C. S. Kley, C. Zhu, D. Kim, S. Lin, C. J. Chang, O. M. Yaghi, P. Yang, *J. Am. Chem. Soc.* **2015**, *137*, 14129- 14135.

[3] B.-X. Dong, S.-L. Qian, F.-Y. Bu, Y.-C. Wu, L.-G. Feng, Y.-L. Teng, W.-L. Liu, Z.-W. Li, *ACS Appl. Energy Mater.* **2018**, *1*, 4662-4669.

[4] M. J. Liu, S. M. Cao, B. Q. Feng, B. X. Dong, Y. X. Ding, Q. H. Zheng, Y. L. Teng, Z. W. Li, W. L. Liu, L. G. Feng, *Dalton Trans* **2020**, *49*, 14995-15001.

[5] Z. Xin,; Y.-R. Wang, Y. Chen, W.-L. Li, L.-Z. Dong, Y.-Q. Lan, *Nano Energy* **2020**, *67*, 104233-104241.

[6] Y. R. Wang, Q. Huang, C. T. He, Y. Chen, J. Liu, F. C. Shen, Y. Q. Lan, *Nat. Commun*. **2018**, *9*, 4466-4473.

Author Contributions

Z. X, Y. C. and Y.-Q. L. conceived and designed the idea. X. D., Y.-R W., Q. W. designed the experiments, collected and analyzed the data. K. S. and J.-W. S. assisted with the experiments and characterizations. Z. X. wrote the manuscript. Y. C. and Y.-Q. L. discussed the results and prepared the manuscript. All the authors reviewed and contributed to this paper.
